# Chemical Structure and Immune Activation of a Glucan From *Rhizoma Acori Tatarinowii*

**DOI:** 10.3389/fnut.2022.942241

**Published:** 2022-06-29

**Authors:** Wuxia Zhang, Jiaqi He, Yihua Hu, Jingwu Lu, Jinzhong Zhao, Peng Li

**Affiliations:** Shanxi Key Laboratory for Modernization of TCVM, Department of Basic Sciences, Shanxi Agricultural University, Shanxi, China

**Keywords:** polysaccharide, structure, immune activity, *Rhizoma Acori Tatarinowii*, glucan

## Abstract

*Rhizoma Acori Tatarinowii* is a traditional Chinese herb used to treat depression and coronary heart disease. Studies on its active components mainly focus on small molecular compounds such as asarone and other essential oil components, while the large molecular active components such as polysaccharides are ignored. In this study, we aimed to study the chemical structure and immune activation of polysaccharides from *Rhizoma Acori Tatarinowii*. In this study, a polysaccharide (RATAPW) was isolated and purified by DEAE-52 cellulose and Sephadex G-100 column chromatography from alkali extraction polysaccharide of *Rhizoma Acori Tatarinowii*. The average molecular weight of RATAPW was 2.51 × 10^4^ Da, and the total carbohydrate contents of RATAPW were 98.23 ± 0.29%. The monosaccharide composition, methylation, and nuclear magnetic resonance (NMR) analysis results displayed that the polysaccharide was α-1,4-glucan with short α-1,6 branches. Immunofluorescence assay and inhibitor neutralization assay indicated that RATAPW could promote the TNF-α production of RAW264.7 macrophage through the nuclear factor kappa B (NF-κB) molecular signaling pathway. Treatment with 200 μg/ml of RATAPW enhanced a 38.77% rise in the proliferation rate of spleen lymphocytes. RATAPW also enhances ConA-induced T cells and lipopolysaccharide (LPS)-induced B cell proliferation in a dose-dependent effect. Our study lays a foundation for the discovery of natural polysaccharide immune modulators or functional food from *Rhizoma Acori Tatarinowii*.

## Introduction

Herb medicine *Rhizoma Acori Tatarinowii* has appetizing, sedative, detumescent, and analgesic effects and is often used to treat joint pain, depression, and Alzheimer’s disease syndrome (1). To date, *Rhizoma Acori Tatarinowii* has been studied for various biological activities, such as potentiating neuronal differentiation of PC12 cells (2), inhibiting the proliferation of cancer cells (3), and promoting the absorption and transport of compounds by inhibiting glycoprotein (4).

However, studies on the active components of *Rhizoma Acori Tatarinowii* mainly focused on the small molecular compounds such as asarone and other essential oil components but ignored the large molecular components such as polysaccharides. In recent years, natural polysaccharides attracted extensive attention for their immunomodulatory activities (5). For example, a polysaccharide from the fruiting bodies of *Helvella leucopus* induced macrophage activation *via* the nuclear factor kappa B (NF-κB) signal pathway (6). The α-glucan from ginger could activate RAW264.7 cells to release cytokines (7).

Inspired by these studies, we speculated that the immune activity of *Rhizoma Acori Tatarinowii* may be related to the active polysaccharide components. Consistently, our previous studies showed that the polysaccharides obtained by water extraction have prominent immunological activity (8). However, the extraction yields, structures, and bioactivities of polysaccharides are strictly affected by the extraction process (9). Studies have shown that polysaccharides obtained by different extraction methods vary greatly. He et al. showed alkalic-extractable polysaccharides from *Ziziphus jujuba cv.* with higher antioxidant activity (10). In this study, we aimed to study the chemical structure and immune activation of polysaccharides from *Rhizoma Acori Tatarinowii*. We extracted the polysaccharide from the *Rhizoma Acori Tatarinowii* with the alkaline solution. The crude polysaccharide was isolated and purified by anion exchange and gel filtration chromatography. The chemical structure was characterized by monosaccharide compositions, infrared spectra (IR), methylation, and nuclear magnetic resonance (NMR) analyses. The immune activation of the polysaccharide was determined by enzyme-linked immunosorbent assay (ELISA), immunofluorescence assay, and inhibitor neutralization assay.

## Materials and Methods

### *Rhizoma Acori Tatarinowii* and Chemical Reagents

Dried herb *Rhizoma Acori Tatarinowii* was produced in Sichuan Province, China. DEAE-52 cellulose, Sephadex G-100, and 3-(4,5-dimethyl-2-triazolyl)-2,5-diphenyltetrazolium bromide (MTT) were purchased from Beijing Solarbio. DAPI, BAY 11-7082, NF-κB p65 antibody, and FITC labeled Goat Anti-Rabbit IgG were supplied by Shanghai Beyotime Biotechnology. ELISA kits were manufactured in R&D Systems.

### Isolation and Purification of Polysaccharide From *Rhizoma Acori Tatarinowii*

The dried *Rhizoma Acori Tatarinowii* was grounded into powder and then defatted with 95% ethanol. The solid residues were extracted with distilled water (1:10, w/v) at 80°C for 2 h, and this extraction process was repeated 3 times. The supernatants were pooled, collected, and studied previously (8). To obtain more kinds of polysaccharides, we further extracted polysaccharides from the residue by alkali extraction and alcohol precipitation. The solid residue was extracted 3 times with 0.2 M sodium hydroxide at 50°C for 2 h. Subsequently, the collected supernatants were concentrated, neutralized, and precipitated with 4 times absolute ethyl alcohol. The polysaccharide precipitates were collected and redissolved in distilled water. Then, the polysaccharide solution was dialyzed for 72 h in distilled water (change the water every 4 h and Mw cutoff: 3,500 Da). After freeze-drying, the alkali extracted crude polysaccharide was obtained. The crude polysaccharide was dissolved in deionized water, and the mixture was centrifuged. The supernatant was loaded on DEAE-52 cellulose (5 cm × 50 cm, Cl^–^ form) and eluted with distilled water and different concentrations of gradient NaCl solution (0.1, 0.2, and 0.5 M NaCl) at a constant flow rate consecutively. The total carbohydrate content of each fraction was detected using the phenol-sulfuric acid method (11). According to the solubility and content of polysaccharides, water elute was further purified using a Sephadex G-100 column. The collected components were eluted with distilled water at a flow rate of 0.2 ml/min. The eluent in the test tube with absorbance greater than 0.3 was collected, lyophilized, and named RATAPW.

### Characterization of Polysaccharides

#### Molecular Weight Measurement

The purity and molecular weight of RATAPW were analyzed by high-performance gel permeation chromatography (HPGPC) performed on three columns (Waters Ultrahydrogel 250, 1,000, and 2,000; 30 cm × 7.8 mm; 6 μm particles) in series (12). The purified RATAPW was eluted with 3 mmol/L sodium acetate at 0.5 ml/min. Notably, 5.2, 11.6, 23.8, 48.6, 148, 273, and 410 kDa dextrans were used as standards. The calibration curve is calculated using Log (Mw) = –0.1719T + 11.585 (T: elution time).

#### Infrared Spectra Analysis

An amount of 2 mg dry RATAPW was mixed with 50 mg chromatographic pure KBr. Agate pestle and mortar were used for grounding the sample, and it was further analyzed using the Fourier transform infrared spectrophotometer (BRUCK, Germany) after pressing in pellets (13). The measurement wavenumber region ranged from 400 cm^–1^ to 4,000 cm^–1^.

#### Chemical Component and Monosaccharide Composition Analysis

The phenol–sulfuric acid method and Bradford’s method (14) were used to determine the total sugar and protein contents, respectively.

An amount of 10 mg RATAPW was hydrolyzed using the 3 M TFA at 120°C for 3 h (15). The hydrolysate was washed three times with methanol and evaporated. Finally, the hydrolyzed material was dissolved with 5 ml of deionized water, transferred to a 50 ml volumetric flask, and diluted to 50 ml. High-performance anion-exchange chromatography (HPAEC) equipped with ICS-5000 (Waltham, MA, United States) and a CarboPac™ PA-20 analytical column (3 mm × 150 mm) was employed. Here, 15 mM NaOH and 100 mM sodium acetate were used as mobile phases for gradient elution at 0.3 ml/min (16). The monosaccharide kinds of RATAPW were determined by comparing the retention times with fifteen monosaccharide standards (fucose, galactosamine, rhamnose, arabinose, glucosamine, galactose, glucose, N-acetyl-D-glucosamine, xylose, mannose, ribose, galacturonic acid, guluronic acid, glucuronic acid, and mannuronic acid).

#### Methylation and Glycosidic Linkage Analysis

The methylation analysis method was applied to confirm the glycosidic linkages of RATAPW based on the reference (17). In brief, the dried polysaccharide RATAPW (3 mg) was dissolved in 1 ml dried DMSO and dried NaOH powder, ultrasonically treated for 1 h. Subsequently, the methyl iodide was added under dark conditions and stirred at 30°C for 1 h to obtain the methylated product. Further hydrolyzed with 4 M TFA, reduced with NaBH_4_, and acetylated with acetic anhydride. Ultimately, the partially methylated alditol acetates (PMAA) containing the linkage type of RATAPW were analyzed by chromatography-mass spectrometry (GC–MS) system (Shimadzu GCMS-QP 2010) equipped with an RXI-5 SIL MS column (30 m × 0.25 mm × 0.25 μm).

#### Nuclear Magnetic Resonance Analysis

A Bruker 600 MHz spectrometer equipped with a ^1^H/^13^C double probe was used for NMR analysis at 25°C (18). An amount of 50 mg dried RATAPW was dissolved in 1 ml D_2_O and frozen three times to exchange H protons into deuterium completely. The lyophilized sample was then dissolved in 0.5 ml D_2_O overnight before NMR analysis. ^1^H NMR, ^13^C NMR, ^1^H-^1^H COSY, HSQC, HMBC, and NOESY spectra of RATAPW were recorded.

### Measurement of TNF-α Cytokine Production Induced by RATAPW

The RAW264.7 cell was a kind gift from Prof. Jinyou Duan at Northwest A&F University. In 96-well plates, RAW 264.7 cells (1 × 10^5^ cells/well) were incubated with different concentrations (50, 100, or 200 μg/ml) of RATAPW for 24 h. phosphate buffer saline (PBS) was used as the negative control and lipopolysaccharide (LPS) (2 μg/ml) was used as the positive control. The cell supernatants were collected. TNF-α proteins in the cell supernatants were measured using the ELISA kits (R&D) according to the instructions (19). To determine the effect of RATAPW on the growth profile of RAW264.7 cells, 0.5 mg/ml MTT was added to the plates and further incubated for 4 h at 37°C. The optical density was measured at 570 nm.

In addition, RAW 264.7 cells were pretreated with PBS or 3 μM BAY 11-7082 for 1 h and incubated with 200 μg/ml RATAPW or LPS (2 μg/ml) for 24 h. TNF-α cytokine content in the different wells was measured using the ELISA kits.

### Immunofluorescence Tests for NF-κB Activation

After being treated with 200 μg/ml RATAPW or LPS (2 μg/ml) for 3 h, the primary NF-κB p65 antibody was added to RAW 264.7 cells for 1 h and then incubated with a FITC-labeled second antibody for 1 h. Finally, we added DAPI and viewed green p65 protein and blue nuclei fluoresce by laser confocal microscopy.

### Effect of RATAPW on Lymphocyte Proliferation

The spleen lymphocyte suspension was obtained by grinding, sieving, and lysis of erythrocytes. Notably, 1 × 10^7^ cells/well lymphocyte cells in 96-well plates were incubated with different concentrations of RATAPW or pre-added the mitotic inducer Con A (5 μg/ml) and LPS (2 μg/ml). After 48 h, the MTT method was used to evaluate the effect of RATAPW on lymphocyte proliferation *in vitro*.

### Statistical Analysis

Dates were expressed as mean value ± standard deviation. One-way and two-way ANOVA were used for statistical significance analysis using the GraphPad Prism 8.0 software.

## Results

### Alkali Extraction and Column Chromatography Purification of Polysaccharide

**FIGURE 1:**
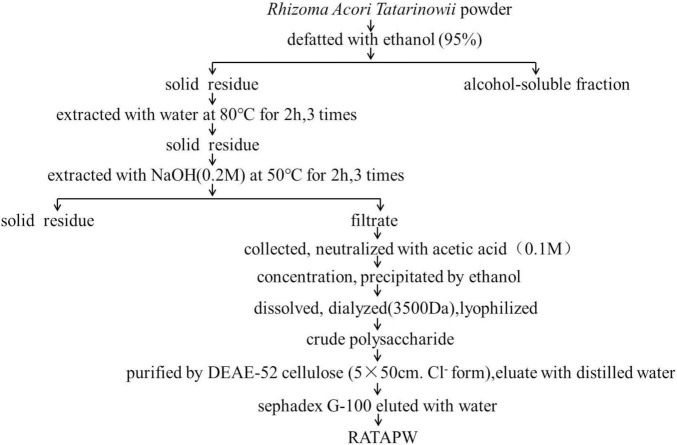
Flowchart of purification polysaccharide RATAPW from *Rhizoma Acori Tatarinowii.*

The crude polysaccharide was isolated from dried *Rhizoma Acori Tatarinowii* through alkali extraction (Figure 1). A polysaccharide designated RATAPW was obtained after DEAE-52 cellulose ion-exchange chromatography and gel filtration chromatography purification steps. The yield of RATAPW was 2.41 ± 0.27% from the crude polysaccharide.

### Molecular Weight and Infrared Spectral Analysis

**FIGURE 2:**
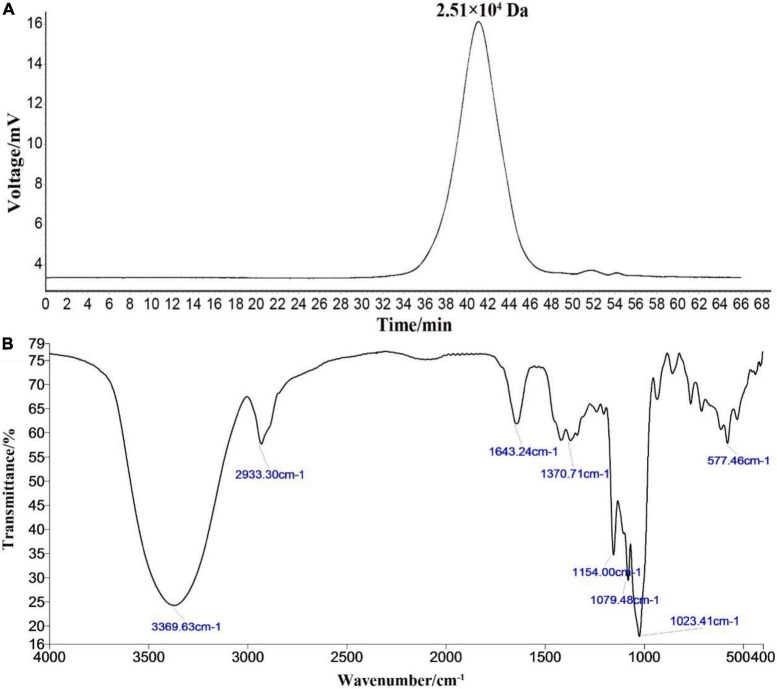
High-performance gel permeation chromatography spectra **(A)** and infrared spectra **(B)** of RATAPW.

The HPGPC peak revealed that the polysaccharide RATAPW was homogeneous and of high purity, with only one symmetrical absorption peak (20). The weight-average molecular weight (Mw) of RATAPW was 2.51 × 10^4^ Da (T: 41.803 min) according to the calibration curve (Figure 2A). In Figure 2B, the bands in the 3,369.63 cm^–1^, 2,933.30 cm^–1^, 1,643.24 cm^–1^, and 1,370 cm^–1^ regions are characteristic absorption peaks of RATAPW (21). The typical vibration at 3,369.63 cm^–1^ corresponded to the -OH stretching. The bands at 2,933.30 cm^–1^ and 1,370 cm^–1^ were the characteristic absorption of C-H stretching vibration and the variable angular vibration of C-H. The absorbance band at 1,643.24 cm^–1^ represented asymmetric stretching vibrations of *C* = O bonds.

### Chemical and Monosaccharide Composition of RATAPW

**FIGURE 3:**
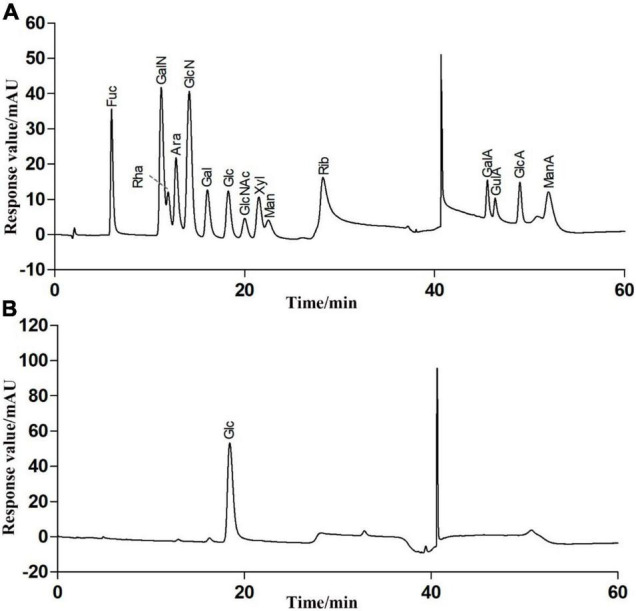
High-performance anion exchange chromatogram of the fifteen mixed standard monosaccharaides **(A)** and purified polysaccharide RATAPW **(B)**.

The total sugar contents in this fraction were 98.23 ± 0.29% after lyophilization. Only a trace amount of protein (1.52 ± 0.07%) was measurable in RATAPW. HPAEC results showed that RATAPW was mainly composed of glucose (Figure 3).

### Glycosyl Linkage Types of RATAPW

After methylation, hydrolysis, and acetylation, the PMAAs of RATAPW were analyzed using gas chromatography-mass spectrometry (GC-MS). The major glycosyl linkage type was →4)-Glcp-(1→ in RATAPW (Table 1). In addition, 2.3% →4,6)-Glcp-(1→, 2.89% →6-Glcp-(1→ and 2.57% non-reducing terminals Glcp-(1→ were also detected. Therefore, the backbone of RATAPW should be 1,4-linked-Glcp, and there are three branches at C-6 for one hundred glucose residues in the backbone.

**TABLE 1:** Linkage type analysis of RATAPW using gas chromatography-mass spectrometry (GC-MS).

RT	Methylated sugar	Mass fragments (m/z)	Area ratios	Type of linkage
16.11	2,3,4,6-Me4-Glcp	43,45,71,87,101,118,129,145,162	2.57	Glcp-(1→
21.231	2,3,6-Me3-Glcp	43,45,87,99,101,113,118,129,131,162,173,233	92.24	→4)-Glcp-(1→
22.265	2,3,4-Me_3_-Glcp	43,45,87,99,101,118,129,162,189,233	2.89	→6-Glcp-(1→
27.192	2,3-Me2-Glcp	43,85,87,99,101,118,127,159,162,201,261	2.3	→4,6)-Glcp-(1→

### Nuclear Magnetic Resonance Analysis

**FIGURE 4:**
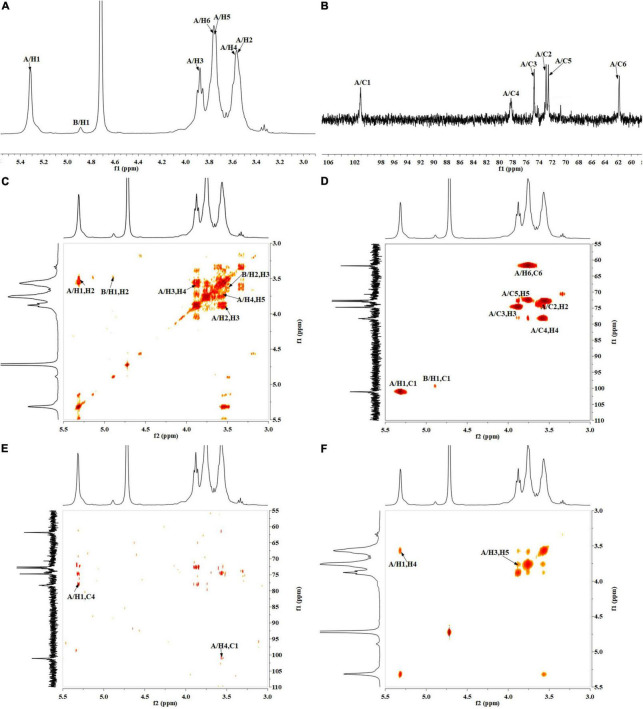
NMR spectra of RATAPW: **(A)** ^1^H NMR, **(B)** ^13^C HMR, **(C)** ^1^H-COSY, **(D)** HSQC, **(E)** HMBC, and **(F)** NOESY. In each figure, codes A and B represent the →4)-α-Glcp-(1→ and α-Glcp-(1→ residue, respectively.

The precise structural information of RATAPW was identified by NMR spectroscopy. The chemical shifts of main glycosyl linkage residues →4)-α-Glcp-(1→ and →6)-α-Glcp-(1→ were assigned (Table 2). The signals at 5.31 and 4.91 ppm in the ^1^H NMR spectrum were attributed to the H1 of →4)-α-Glcp-(1→ and α-Glcp-(1→ (Figure 4A). The overlapping broad peaks around 5.31 ppm indicated the existence of branches in →4)-α-Glcp-(1→ residue. According to the literature (22), the main signal at δ 101.05 ppm was assigned to the C1 of →4)-α-Glcp-(1→ in ^13^C NMR (Figure 4B).

**TABLE 2:** ^1^H NMR and ^13^C NMR spectral assignments for RATAPW.

Glycosyl residues	H1	H2	H3	H4	H5	H6a	H6b
		
	C1	C2	C3	C4	C5	C6	
→4)-α-Glcp-(1→	5.31	3.55	3.9	3.58	3.78	3.79	3.71
	101.05	72.91	74.56	78.31	72.53	61.89	
α-Glcp-(1→	4.91	3.53	3.67	–	–	–	–
	99.1	–	–	–	–	–	–

The other signals of H/C were analyzed by COSY and HSQC spectrum (Figures 4C,D). In ^1^H–^1^H COSY, the cross-peaks at δH/H 5.31/3.55, 3.55/3.90, 3.90/3.58, and 3.58/3.78 ppm suggested that the signals at δ 5.31, 3.55, 3.65, 3.90, and 3.58 ppm corresponded to H1, H2, H3, H4, and H5 of →4)-α-Glcp-(1→ (residue **A**), respectively. HSQC showed that the H1 and C1 signals of →4)-α-Glcp-(1→ were 101.05 and 5.31 ppm. C1–C6 of the residue →4)-α-Glcp-(1→ were δ 101.05, 72.91, 74.56, 78.31, 72.53, and 61.89 ppm, respectively. In the HMBC spectrum (Figure 4E), cross-peaks at δ 5.31/78.31 and δ 3.58/101.05 ppm represented the correlation between H1/C4 and H4/C1 of residue →4)-α-Glcp-(1→, which suggested that the backbone of RATAPW was →4)-α-Glcp-(1→4)-α-Glcp-(1→.

**FIGURE 5:**
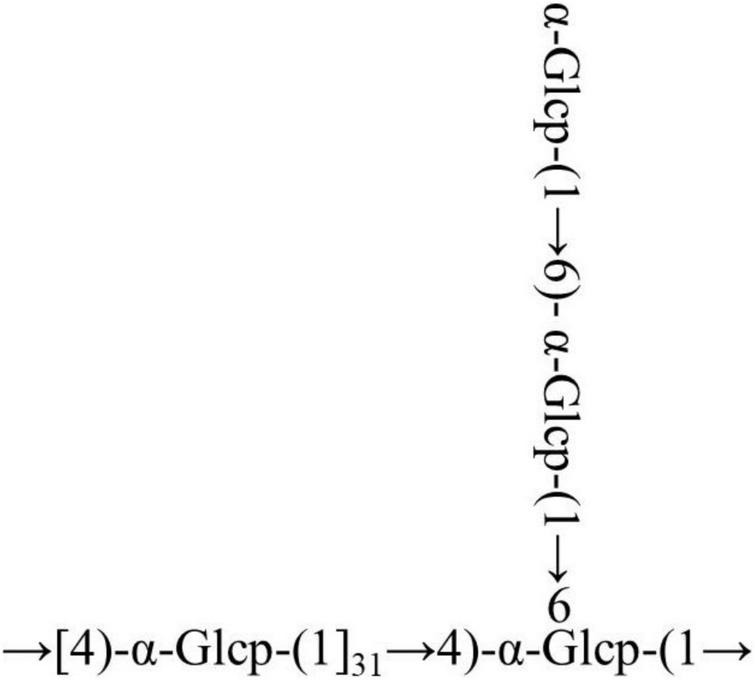
Putative structure of RATAPW.

The cross-peaks of H1/H4 and H3/H5 in the NOESY spectrum also indicate that the →4)-α-Glcp-(1→ residue was alpha configuration (Figure 4F). Based on the monosaccharide composition, methylation, and NMR analysis results, the possible structure of RATAPW was a 1,4-linked α-glucans and branched at C-6 every 32 →4)-α-Glcp-(1→ residue (Figure 5).

### RATAPW Induced RAW 264.7 to Produce TNF-α *via* the NF-κB Pathway

**FIGURE 6:**
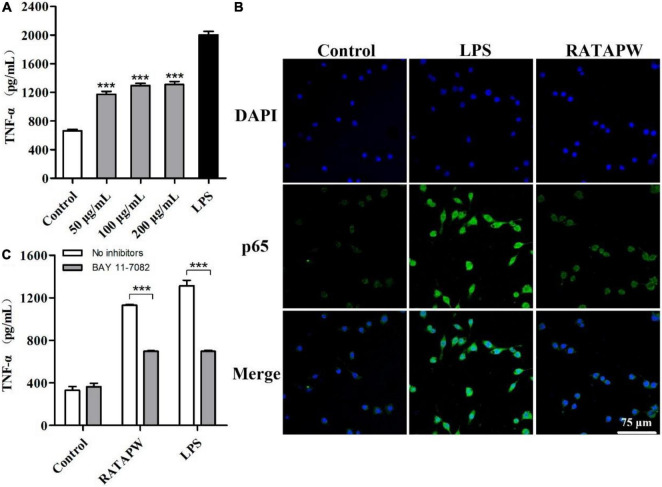
RATAPW activates macrophages *via* the NF-κB pathway. **(A)** RAW264.7 cells were incubated with RATAPW for 24 h, and TNF-α production was tested. ***p < 0.001 were compared with the control group. **(B)** An amount of 200 μg/ml RATAPW or 2 μg/ml lipopolysaccharide (LPS) were added to RAW264.7 cells for 3 h, and p65 protein (green) in the nuclei (DAPI) was determined by laser confocal microscopy. **(C)** After being pretreated with PBS (no inhibitors) or BAY 11-7082 for 1 h, RAW264.7 cells were incubated with 200 μg/ml RATAPW or 2 μg/ml LPS for another 24 h. TNF-α production in the supernate was tested.

The MTT results showed that different concentrations of RATAPW did not promote the proliferation of macrophages (Supplementary Figure 1). As shown in Figure 6A, TNF-α production of RAW 264.7 cells was significantly increased by RATAPW with a dose-dependent effect. Endotoxin contamination results showed that the endotoxin content is less than 0.01 EU, which ensures that the effect of RATAPW on RAW 264.7 cells was not due to endotoxin contamination (23).

Nuclear factor kappa B is a pivotal nuclear transcription factor, which is associated with multiple immune genes and manipulated the cytokine responses (24). External irritants can cause the inactive IκBα in the cytosolic phosphorylation and degradation and then induce p65 subunit translocation to the nucleus (25, 26). Thus, we detected the NF-κB activation in RAW264.7 cells by immunofluorescence assay. Results showed that RATAPW and LPS could activate NF-κB and make cytoplasmic NF-κB p65 protein translocated to the nucleus (Figure 6B). Furthermore, to validate whether the NF-κB activation was involved in RATAPW-induced cytokine production, 3 μM BAY 11-7082 (an NF-κB inhibitor) was added before adding RATAPW or LPS. Experimental results showed that BAY 11-7082 overtly suppressed TNF-α secretion in RAW264.7 cells (Figure 6C). All these results show that RATAPW can activate macrophages *via* the NF-κB pathway.

### RATAPW Promotes Splenocyte Proliferation

**FIGURE 7:**
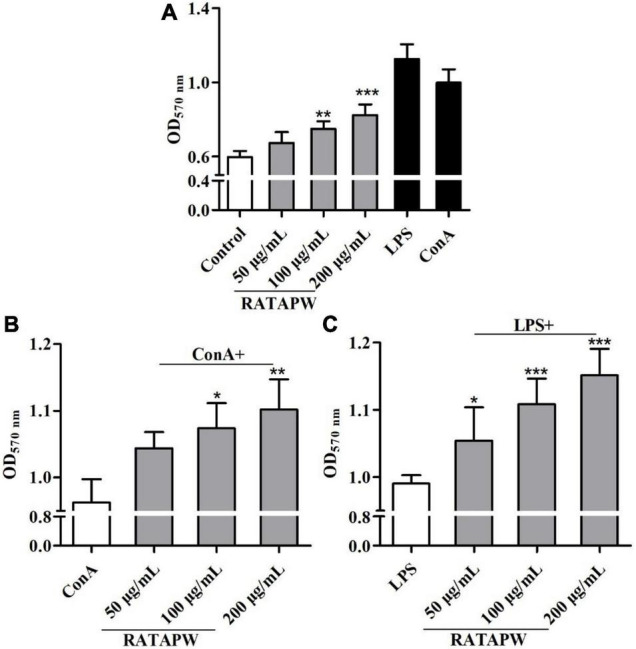
RATAPW promoted splenocyte proliferation. Different concentrations of RATAPW **(A)**, in the presence of mitogens Con A (5 μg/ml) **(B)** or LPS (2 μg/ml) **(C)** were added to the spleen cells for 48 h, and the number was determined using 3-(4,5-dimethyl-2-triazolyl)-2,5- diphenyltetrazolium bromide (MTT) method. ***p < 0.001, **p < 0.01, and *p < 0.05 were compared with the control group **(A)**, ConA group **(B)**, or LPS group **(C)**, respectively.

As a major site of immune responses, the induction and regulation of spleen proliferation are very important for the immune system (27). Mouse primary splenocytes are mainly composed of T and B cells (28). Our results showed that RATAPW promoted the proliferation of spleen lymphocytes (Figure 7A). It also acted in concert to synergistically enhance ConA-stimulated T cells and LPS-induced B cell proliferation in a dose-dependent effect (Figures 7B,C).

## Discussion

In this study, a polysaccharide named RATAPW (molecular weight: 2.51 × 10^4^ Da) was isolated from *Rhizoma Acori Tatarinowii* with alkali extraction and alcohol precipitation, and its primary chemical structure, physicochemical properties, and immune activity were characterized. The HPAEC results showed that RATAPW was mainly composed of glucose, combined with methylation analysis results and NMR spectra, and RATAPW was an α-1,4-glucans branched at C-6 every 32 glucose residues. The bioactivity tests showed that RATAPW could activate the RAW 264.7 cells increasing TNF-α secretion by NF-κB pathways. Besides, RATAPW can also enhance T and B lymphocyte proliferation.

Glucans are divided into α- and β-conformation, and the linkage of the glucose can be either 1,4 or 1,6 glycoside bonds (29). Reports suggested that the chemical structure and chain conformation of glucan directly affect its biological activity (30). Therefore, it is necessary to detail the structure-activity relationships between glucans and immunoregulation. Till now, it has been known that many natural β-glucans exhibit significant immunomodulatory activities (31). A set of receptors for β-glucans have been revealed, such as toll-like receptors (TLRs), dectin-1, complement receptor 3 (CR3), and lactosylceramide (30). Similarly, a lot of studies have demonstrated that α-glucans also had kinds of immune activities, which can be directly influenced by the solubility, molecular weight, molecular charge, branching degree, and glycosidic bonds of α-glucans (32).

Reports suggest that the (1→3)-, (1→4)-, or (1→6)-α-glucans can stimulate immune cells to secrete cytokines to different degrees (33). Consistently, our results showed that RATAPW was an α-1,4-glucan with few (1→6) branches and can activate macrophages to release cytokines. Our previous study has displayed that two α-glucans from *Radix Paeoniae Alba* with a similar structure to RATAPW can also promote splenocyte proliferation and RAW264.7 phagocytic activity (34). The molecular weight is another important impact factor for macrophage stimulation and the production of cytokines. The molecular weight of RATAPW was 25.1 kDa, within the range of 10 kDa to 1,000 kDa. Previous reports have indicated that polysaccharides with molecular weights in this range have the highest immunoregulatory activities (35). The solubility can also affect the recognition of polysaccharides by the antigen-presenting cells (APCs) and cytokine production (36). It has been demonstrated that the increase of water solubility of α-glucans enhances its immunostimulant activity (37). The immune activity of RATAPW might also be linked to its high solubility. Therefore, it was speculated that the immunoregulatory activity of RATAPW might be attributed to its high solubility, moderate molecular weight, and low degree of branching.

Glucans are toll-like receptor 4 (TLR4) ligands on the macrophages’ cell surface and activate immune cells through the TLR4/IKK/NK-κB pathway (38). α-Glucan could also activate the MAPK signaling pathway to promote the secretion of cytokines (9). Our results proved that RATAPW activates the RAW 264.7 cells and increases TNF-α secretion by the NF-κB pathways. The possible receptor and molecular pathway of RATAPW need to be further explored. In general, the glucan RATAPW could be explored as an immunomodulator in the field of health food and medicine.

## Data Availability Statement

The original contributions presented in this study are included in the article/Supplementary Material, further inquiries can be directed to the corresponding authors.

## Author Contributions

WZ designed the research, performed the experiments, analyzed the data, and wrote the manuscript. JH and YH performed the experiments. JL analyzed the experimental data. JZ analyzed the NMR data. PL supervised the work and wrote and reviewed the manuscript. All authors contributed to the article and approved the submitted version.

## Conflict of Interest

The authors declare that the research was conducted in the absence of any commercial or financial relationships that could be construed as a potential conflict of interest.

## Publisher’s Note

All claims expressed in this article are solely those of the authors and do not necessarily represent those of their affiliated organizations, or those of the publisher, the editors and the reviewers. Any product that may be evaluated in this article, or claim that may be made by its manufacturer, is not guaranteed or endorsed by the publisher.

## Abbreviations

MTT: 3-(4,5-dimethyl-2-triazolyl)-2,5-diphenyltetrazolium bromide
ConA: concanavalin A
BAY 11-7082: (E)-3-[(4-methylphenylsulfonyl]-2-propenenitrile
LPS: lipopolysaccharide
DAPI: 2- (4-Amidinophenyl)-6-indolecarbamidine dihydrochloride
TFA: trifluoroacetic acid
DMSO: dried dimethyl sulfoxide
ELISA: enzyme-linked immunosorbent assay
PMAA: partially methylated alditol acetates
APC: the antigen-presenting cell
MAPKs: mitogen-activated protein kinases.

## References

[1] Fu Y Yang Y Shi J Bishayee K Lin L Lin Y Acori tatarinowii rhizoma extract ameliorates Alzheimer’s pathological syndromes by repairing myelin injury and lowering Tau phosphorylation in mice. *Pharmazie.* (2020) 75:395–400. 10.1691/ph.2020.0492 32758340

[2] Lam K Huang Y Yao P Wang H Dong T Zhou Z Comparative study of different *Acorus* species in potentiating neuronal differentiation in cultured PC12 cells. *Phytother Res.* (2017) 31:1757–64. 10.1002/ptr.5904 28833752

[3] Wu J Zhang X Sun Q Chen M Liu S Zhang X β-asarone inhibits gastric cancer cell proliferation. *Oncol Rep.* (2015) 34:3043–50. 10.3892/or.2015.4316 26502896

[4] Shi B Liu J Zhang Q Wang S Jia P Bian L Effect of co-administration of Acori Tatarinowii Rhizoma volatile oil on pharmacokinetic fate of xanthotoxol, oxypeucedanin hydrate, and byakangelicin from *Angelicae dahuricae* Radix in rat. *J Sep Sci.* (2020) 43:2349–62. 10.1002/jssc.201901250 32222035

[5] Li Y Wang X Ma X Liu C Wu J Sun C. Natural polysaccharides and their derivates: a promising natural adjuvant for tumor immunotherapy. *Front Pharmacol.* (2021) 12:621813. 10.3389/fphar.2021.621813 33935714 PMC8080043

[6] Zhang W Gong L Zhou Z Sun M Li Y Sun J Structural characterization and immunomodulatory activity of a mannan from *Helvella leucopus*. *Int J Biol Macromol.* (2022) 212:495–507. 10.1016/j.ijbiomac.2022.05.132 35618090

[7] Yang X Wei S Lu X Qiao X Simal-Gandara J Capanoglu E A neutral polysaccharide with a triple helix structure from ginger: characterization and immunomodulatory activity. *Food Chem.* (2021) 350:129261. 10.1016/j.foodchem.2021.129261 33610845

[8] Zhang W Song D Xu D Wang T Chen L Duan J. Characterization of polysaccharides with antioxidant and immunological activities from Rhizoma Acori Tatarinowii. *Carbohydr Polym.* (2015) 133:154–62. 10.1016/j.carbpol.2015.07.018 26344267

[9] Cui F Jiang L Qian L Sun W Tao T Zan X A macromolecular α-glucan from fruiting bodies of *Volvariella volvacea* activating RAW264. 7 macrophages through MAPKs pathway. *Carbohydr Polym.* (2020) 230:115674. 10.1016/j.carbpol.2019.115674 31887864

[10] He Z Zhu Y Bao X Zhang L Li N Jiang G Optimization of alkali extraction and properties of polysaccharides from *Ziziphus jujuba* cv. residue. *Molecules.* (2019) 24:2221. 10.3390/molecules24122221 31197074 PMC6631402

[11] DuBois M Gilles K Hamilton J Rebers P Smith F. Colorimetric method for determination of sugars and related substances. *Anal Chem.* (2002) 28:350–6.

[12] Zhang W Chen L Li P Zhao J Duan J. Antidepressant and immunosuppressive activities of two polysaccharides from Poria cocos (Schw.) Wolf. *Int J Biol Macromol.* (2018) 120:1696–704. 10.1016/j.ijbiomac.2018.09.171 30267822

[13] Kakar M Li J Mehboob M Sami R Benajiba N Ahmed A Purification, characterization, and determination of biological activities of water-soluble polysaccharides from *Mahonia bealei*. *Sci Rep.* (2022) 12:8160. 10.1038/s41598-022-11661-3 35581215 PMC9114413

[14] Bradford M. A rapid and sensitive method for the quantitation of microgram quantities of protein utilizing the principle of protein-dye binding. *Anal Biochem.* (1976) 72:248–54. 10.1006/abio.1976.9999 942051

[15] Wang Z Liu X Bao Y Wang X Zhai J Zhan X Characterization and anti-inflammation of a polysaccharide produced by *Chaetomium globosum* CGMCC 6882 on LPS-induced RAW 264.7 cells. *Carbohydr Polym.* (2021) 251:117129. 10.1016/j.carbpol.2020.117129 33142660

[16] Wang Z Xue R Cui J Wang J Fan W Zhang H Antibacterial activity of a polysaccharide produced from *Chaetomium globosum* CGMCC 6882. *Int J Biol Macromol.* (2019) 125:376–82. 10.1016/j.ijbiomac.2018.11.248 30500504

[17] Ji X Guo J Ding D Gao J Hao L Guo X Structural characterization and antioxidant activity of a novel high-molecular-weight polysaccharide from *Ziziphus Jujuba* cv. Muzao. *J Food Meas Character.* (2022) 16:2191–200. 10.1007/s11694-022-01288-3

[18] Ji X Guo J Pan F Kuang F Chen H Guo X Structural elucidation and antioxidant activities of a neutral polysaccharide from Arecanut (*Areca catechu* L.). *Front Nutr.* (2022) 9:853115. 10.3389/fnut.2022.853115 35340550 PMC8948432

[19] Zhang W Hu Y He J Guo D Zhao J Li P. Structural characterization and immunomodulatory activity of a novel polysaccharide from Lycopi Herba. *Front Pharmacol.* (2021) 12:691995. 10.3389/fphar.2021.691995 34248640 PMC8267152

[20] Ji X Cheng Y Tian J Zhang S Jing Y Shi M. Structural characterization of polysaccharide from jujube (*Ziziphus jujuba* Mill.) fruit. *Chem Biol Technol Agric.* (2021) 8:54. 10.1186/s40538-021-00255-2

[21] He P Dong Z Wang Q Zhan QP Zhang MM Wu H. Structural characterization and immunomodulatory activity of a polysaccharide from *Eurycoma longifolia*. *J Nat Prod.* (2019) 82:169–76. 10.1021/acs.jnatprod.8b00238 30714735

[22] Hu X Huang Y Dong Q Song L Yuan F Yu R. Structure characterization and antioxidant activity of a novel polysaccharide isolated from pulp tissues of Litchi chinensis. *J Agric Food Chem.* (2011) 59:11548–52. 10.1021/jf203179y 21973186

[23] Zhang W Mu H Dong D Wang D Zhang A Duan J. Alteration in immune responses toward N-deacetylation of hyaluronic acid. *Glycobiology.* (2014) 24:1334–42. 10.1093/glycob/cwu079 25091818

[24] Cao L Li R Chen X Xue Y Liu D. Neougonin a inhibits lipopolysaccharide-induced inflammatory responses *via* downregulation of the NF-kB signaling pathway in RAW 264.7 macrophages. *Inflammation.* (2016) 39:1939–48. 10.1007/s10753-016-0429-9 27581278

[25] Yan J Han Z Qu Y Yao C Shen D Tai G Structure elucidation and immunomodulatory activity of a β-glucan derived from the fruiting bodies of *Amillariella mellea*. *Food Chem.* (2018) 240:534–43. 10.1016/j.foodchem.2017.07.154 28946308

[26] Mendes Sdos S Candi A Vansteenbrugge M Pignon MR Bult H Boudjeltia KZ Microarray analyses of the effects of NF-kappaB or PI3K pathway inhibitors on the LPS-induced gene expression profile in RAW264.7 cells: synergistic effects of rapamycin on LPS-induced MMP9-overexpression. *Cell Signal.* (2009) 21:1109–22. 10.1016/j.cellsig.2009.02.025 19285553

[27] Batista F Harwood N. The who, how and where of antigen presentation to B cells. *Nat Rev Immunol.* (2009) 9:15–27. 10.1038/nri2454 19079135

[28] Huang Y Tsai K Tan S Kang S Ford M Harder K 2B4-SAP signaling is required for the priming of naive CD8(+) T cells by antigen-expressing B cells and B lymphoma cells. *Oncoimmunology.* (2017) 6:e1267094. 10.1080/2162402x.2016.1267094 28344876 PMC5353922

[29] Wagener J Striegler K Wagener N. α- and β-1,3-glucan Synthesis and Remodeling. *Curr Top Microbiol Immunol.* (2020) 425:53–82. 10.1007/82_2020_200 32193600

[30] Jin Y Li P Wang F. β-glucans as potential immunoadjuvants: a review on the adjuvanticity, structure-activity relationship and receptor recognition properties. *Vaccine.* (2018) 36:5235–44. 10.1016/j.vaccine.2018.07.038 30049632

[31] Córdova-Martínez A Caballero-García A Roche E Noriega DC. β-glucans could be adjuvants for SARS-CoV-2 virus vaccines (COVID-19). *Int J Environ Res Public Health.* (2021) 18:12636. 10.3390/ijerph182312636 34886361 PMC8656611

[32] Moreno-Mendieta S Guillén D Hernández-Pando R Sánchez S Rodríguez-Sanoja R. Potential of glucans as vaccine adjuvants: a review of the α-glucans case. *Carbohydr Polym.* (2017) 165:103–14. 10.1016/j.carbpol.2017.02.030 28363529

[33] Koppada R Norozian FM Torbati D Kalomiris S Ramachandran C Totapally BR. Physiological effects of a novel immune stimulator drug, (1,4)-α-D-glucan, in rats. *Basic Clin Pharmacol Toxicol.* (2009) 105:217–21. 10.1111/j.1742-7843.2009.00383.x 19389049

[34] Zhang W Li P Song D Niu H Shi S Wang S Structural characterization and biological activities of two α-glucans from radix paeoniae alba. *Glycoconj J.* (2016) 33:147–57. 10.1007/s10719-015-9647-x 26747055

[35] Zhang X Qi C Guo Y Zhou W Zhang Y. Toll-like receptor 4-related immunostimulatory polysaccharides: primary structure, activity relationships, and possible interaction models. *Carbohydr Polym.* (2016) 149:186–206. 10.1016/j.carbpol.2016.04.097 27261743

[36] Wismar R Brix S Laerke HN Frøkiaer H. Comparative analysis of a large panel of non-starch polysaccharides reveals structures with selective regulatory properties in dendritic cells. *Mol Nutr Food Res.* (2011) 55:443–54. 10.1002/mnfr.201000230 20938988

[37] Bao X Duan J Fang X Fang J. Chemical modifications of the (1→3)-alpha-D-glucan from spores of *Ganoderma lucidum* and investigation of their physicochemical properties and immunological activity. *Carbohydr Res.* (2001) 336:127–40. 10.1016/s0008-6215(01)00238-5 11689183

[38] Złotko K Wiater A. A Report on Fungal (1→3)-α-d-glucans: properties. *Funct Appl.* (2019) 24:3972. 10.3390/molecules24213972 31684030 PMC6864487

