# Supplementary material for: Chemical Structure and Immune Activation of a Glucan From Rhizoma Acori Tatarinowii
Source: Front Nutr. 2022 Jun 29;9:942241. doi: 10.3389/fnut.2022.942241 (PMC9277461; doi:10.3389/fnut.2022.942241)
Supplement: Supplementary file 1 [file Image_1.pdf]

Chemical structure and immune activation of a glucan from *Rhizoma Acori Tatarinowii*

Wuxia Zhang*, Jiaqi He, Yihua Hu, Jingwu Lu, Jinzhong Zhao, Peng Li*

Shanxi key lab. for modernization of TCVM, Department of Basic Sciences, Shanxi Agricultural University, Taigu, 030801, Shanxi, China

*Corresponding author:

Wuxia Zhang: E-mail: wuxia200758@163.com

Peng Li: E-mail: lipengcuc@163.com

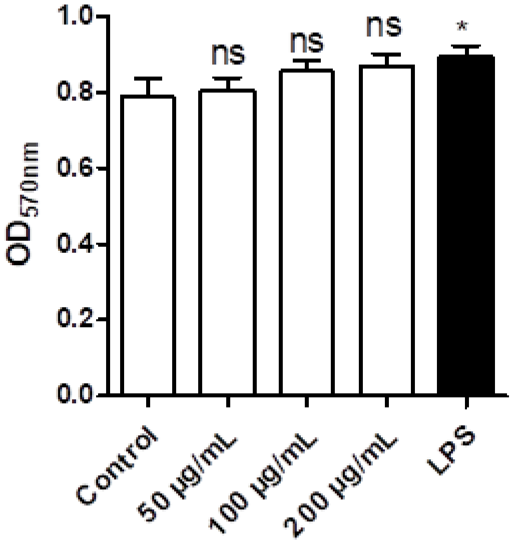

Fig. S1. The effect of RATAPW on the growth profile of RAW264.7 cells. RAW264.7 cells were incubated with different concentrations RATAPW (50, 100, 200 µg/mL) and LPS (2 µg/mL) for 24 h, then 0.5mg/mL MTT was added to the plates and further incubated for 4 h at 37 °C. The optical density was measured at 570 nm. PBS was used as negative control. "*****" represents a significant difference < 0.05 compared with the control group. "**ns**" represents no significant difference compared with the control group.
